# Transcutaneous oxygen pressure as a predictor for short-term survival in patients with type 2 diabetes and foot ulcers: a comparison with ankle–brachial index and toe blood pressure

**DOI:** 10.1007/s00592-018-1145-8

**Published:** 2018-04-30

**Authors:** K. Fagher, P. Katzman, M. Löndahl

**Affiliations:** 1 Clinical Sciences in Lund, Lund University, Lund, Sweden; 2 Department of Endocrinology, Skåne University Hospital, 22185 Lund, Sweden

**Keywords:** Diabetic foot ulcers, Complications, Microvascular disease, Macrovascular disease

## Abstract

**Aims:**

Ankle–brachial index (ABI) is the most commonly used test when diagnosing peripheral vascular disease and is considered a marker for cardiovascular risk. Transcutaneous oxygen pressure (TcPO_2_), a test associated with microvascular function, has in several studies shown better correlation with diabetic foot ulcer (DFU) healing. Whether a low TcPO_2_ could be a marker for mortality in the high-risk population of DFU patients has not been evaluated before. The aim of this study was to evaluate the predictive value of TcPO_2_ in comparison with ABI and toe blood pressure (TBP) on 1-year mortality in type 2 diabetes patients with DFU.

**Methods:**

Type 2 diabetes patients aged ≤ 90 years, with one DFU who attended our multidisciplinary DFU-unit during year 2013–2015 and were screened with TcPO_2_, ABI and TBP were retrospectively evaluated. One-year mortality was assessed from the national death register in Sweden.

**Results:**

A total of 236 patients (30% women) with a median age of 76 (69–82) years were evaluated in this study. Within 1 year, 14.8% of the patients died. TcPO_2_ < 25 mmHg was associated with a higher 1-year mortality compared with TcPO_2_ ≥ 25 mmHg (27.7 vs. 11.6%, *p* = 0.003). TBP and ABI did not significantly influence 1-year mortality. In a Cox regression analysis adjusted for confounders, TcPO_2_ was independently predicting 1-year mortality with a hazard ratio for TcPO_2_ < 25 mmHg of 2.8 (95% CI 1.34–5.91, *p* = 0.006).

**Conclusions:**

This study indicates that a low TcPO_2_ is an independent prognostic marker for 1-year mortality among patients with type 2 diabetes and DFU.

## Introduction

Diabetic foot ulcers (DFU) are a common complication of diabetes mellitus, associated with both an increased risk of amputations in the lower limb and a higher risk of cardiovascular disease and death [1–3]. Peripheral vascular disease (PVD) is frequently contributing, together with peripheral neuropathy in the development of a DFU, and the presence of PVD is considered a predictor of worse outcome both for ulcer healing and survival [4, 5]. Ankle–brachial index (ABI) is the most commonly used test for the diagnosis of PVD and has also been associated with increased cardiovascular risk in the general population [6]. However, in patients with diabetes, both the diagnostic and the predictive value of ABI may be limited due to a high prevalence of false-negative values as a result of medial artery calcification [7–9]. Further, ABI does not reflect microvascular dysfunction, a condition often seen in patients with DFU. Transcutaneous oxygen pressure (TcPO_2_) is a non-invasive method measuring tissue perfusion and is considered to better reflect the microvascular status in the skin. The International Working Group on the Diabetic Foot (IWGDF) recommend in their guideline document, urgent vascular imaging and if feasible revascularisation when TBP < 30 mmHg or TcPO_2_ < 25 mmHg, as patients with higher levels are more likely to heal their ulcers [10]. It has previously been demonstrated a plausible association between TcPO_2_ and mortality in patients with diabetes, but without significant PVD, or ongoing DFUs [11]. Also, in a study by Gazzaruso et al. [12], a low TcPO_2_ has been considered a risk factor for major cardiovascular events (MACEs) in patients with type 2 diabetes, but without a history of foot ulcer, or previous cardiovascular disease. Whether a low TcPO_2_ could be a marker for mortality in the high-risk population of DFU patients has not been evaluated before. The aim of this study was to evaluate the predictive value of TcPO_2_, in comparison with ABI and TBP, on 1-year all-cause mortality in patients with type 2 diabetes and DFU.

## Methods

For this study, we retrospectively enrolled patients with type 2 diabetes, aged ≤ 90 years, with at least one DFU who visited our DFU-unit between year 2013–2015. All patients were examined with TcPO_2_, ABI and TBP, measured with Periflux System 5000 diagnostic instrument (Perimed AB, Stockholm Sweden). These non-invasive assessments are routine in all referrals at our DFU unit.

TcPO_2_-measurements were performed at the dorsum of both feet, while patient was breathing ambient air, in a resting supine position at room temperature, between 21 °C and 24 °C. The site on the foot was carefully cleaned before the transducer was applied to the skin, using adhesive rings and contact liquid, supplied by the manufacturer. The measurement was performed after calibration and preheating of the transducer to approximate 44 °C. Patients were stratified according to TcPO_2_ < and ≥ 25 mmHg, and to evaluate mortality related to different TcPO_2_ levels, patients were also grouped according to TcPO_2_ quartiles.

The systolic ankle pressure and TBP were also evaluated during resting, in a supine position. Three measurements were performed on each foot and averaged. ABI was calculated by dividing the systolic ankle pressure with the systolic arm pressure, and ABI 0.9–1.3 was considered normal [13]. TBP was measured at the great toe, and TBP < 30, as well as < 50 mmHg, was used in the analyses. The lowest value (of ABI, TBP and TcPO_2_) of the two legs was used in mortality analysis, while the value of the affected foot was used when analysing ulcer healing.

Baseline characteristics were assessed from patient’s medical records and included age, gender, diabetes duration, as well as co-morbidities, such as history of hypertension, hyperlipidaemia, cardiovascular disease (CVD), as well as concomitant medication, and laboratory data (HbA_1c_, LDL-cholesterol and creatinine). CVD was defined as verified myocardial infarction, angina pectoris, heart failure or cerebral vascular disease. Hypertension was defined as blood pressure ≥ 140/90, or ongoing treatment with antihypertensive drugs. Hyperlipidaemia was defined as LDL > 2.5 mmol/l, or the ongoing treatment with cholesterol-lowering drugs. Glomerular filtration rate (eGFR) was estimated from plasma creatinine, using the modification of diet in renal disease (MDRD) equation [14].

All patients were treated according to international guidelines, at our multidisciplinary diabetic foot clinic, and were evaluated for vascular intervention when indicated. Ulcer healing, defined as complete epithelialisation within 12 weeks, and above-ankle amputation during follow-up were assessed from patients’ charts. Mortality data were obtained from the National Death Registry of Sweden. Approval of the study was given by the Ethics Committee in Lund, Sweden.

## Statistical analysis

Continuous data are expressed as median and interquartile ranges (IQR; 25–75 percentile), and to assess differences Mann–Whitney *U* tests were performed. Categorical data are expressed as percentages, and Fisher’s exact test was used to compare differences. Survival analyses were performed with Kaplan–Meier estimates, and significances calculated with log-rank tests. A Cox regression analysis was performed, to adjust for confounding factors. Those factors with a significant (*p* < 0.05), or nearly significant (*p* < 0.1) difference between groups at baseline, as well as those with a significant association with mortality in the univariate analysis, were stepwise entered in a multivariate Cox model. Only those variables that changed the *p* value of our variables of interest, or significantly predicted mortality, were kept in the final model. The results of the Cox analysis are given as Hazard ratios (HRs) with 95% confidence interval (CI). All statistical analyses were performed using SPSS program (IBM, version 22). A two-sided *p* value < 0.05 was taken as statistical significant.

## Results

We enrolled 236 type 2 diabetes patients with DFU, with a median age of 76 years (69–82), visiting our multidisciplinary diabetic foot clinic between year 2013 and 2015. A total number of 47 patients had a baseline TcPO_2_ < 25 mmHg, and among them, the probability for healing was low and only 8.8% successfully healed their ulcers after 12 weeks. Further, these patients suffered an increased risk of above-ankle amputations (23.4 vs. 4.2%, in TcPO_2_ ≥ 25 mmHg, *p* = 0.001). Minor amputation or auto-amputation was performed in 17 (9.0%) patients during follow-up in the group with TcPO_2_ ≥ 25 mmHg, compared to 6 (12.8%) in the TcPO_2_ < 25 mmHg group (n.s). Baseline characteristics, as well as healing, and major amputation rates of the study population, and of patients stratified by TcPO_2_ < and ≥ 25 mmHg, are given in Table 1. ABI < 0.9 or > 1.3 was significantly associated with worse ulcer outcome (12-week healing rate of 15.7 vs. 32.7%, in patients with normal ABI, *p* = 0.004), but no significant association between ulcer healing and TBP was found (both < 30 mmHg and < 50 mmHg analysed). Within the population of patients with TcPO_2_ < 25 mmHg, the revascularisation rate during follow-up was 25.5%. Additional 21.3% were vascular assessed, but intervention was not manageable, a decision made by a vascular surgeon. The remaining 53.2% (*n* = 25) of patients in TcPO_2_ < 25 mmHg were separately evaluated concerning plausible explanations why vascular diagnostics or revascularisation was not performed (Table 2).

**Table 1:** Baseline characteristics of all patients and patients stratified according to TcPO_2_ < 25 mmHg

	All patients	TcPO_2_ < 25 mmHg	TcPO_2 _≥ 25 mmHg	*p* value
*n*	236	47	189	
Age (years)	76 (69–82)	74 (68–81)	77 (69–82)	n.s
Sex (male/female %)	69.9/30.1	57.4/42.6	73.0/27.0	0.050
Diabetes duration (years)	15 (8–23)	18 (8–26)	15 (8–22)	n.s
HbA1c (mmol/mol)	59 (50–70)	60 (48–75)	59 (49–68)	n.s
HbA1c (%) (DCCT)	7.5 (6.7–8.6)	7.6 (6.5–9.0)	7.5 (6.6–8.4)	n.s
eGFR (ml min^−1^ 1.73 m^2^)	60 (42–81)	54 (38–68)	63 (43–87)	0.009
Previous CVD	65.3	70.2	64.0	n.s
Antidiabetic treatment				
Insulin (%)	69.8	78.3	67.7	n.s
Sulphonylurea (%)	10.6	6.4	11.7	n.s
Incretins (%)	7.2	10.6	6.4	n.s
SGLT2 inhibitors (%)	0.9	0.0	1.1	n.s
Metformin (%)	38.0	34.0	39.0	n.s
ACEI/ARBs (%)	66.1	70.2	65.1	n.s
Hypertension (%)	94.5	93.6	94.7	n.s
Hyperlipidemia (%)	87.1	86.4	87.3	n.s
Smoking (ever) (%)	26.2	35.0	24.1	n.s
ABI	0.9 (0.6–1.1)	0.7 (0.5–1.0)	0.9 (0.6–1.1)	0.016
TBP (mmHg)	65 (45–93)	45 (26–79)	70 (49–100)	<0.001
TcPO_2_ (mmHg)	41 (28–52)	11 (5–22)	44 (37–54)	
Ulcer healed within 3 months (%)	23.1	8.8	25.5	0.045
Revascularisation during follow-up (%)	16.1	25.5	13.8	0.073
Vascular intervention not possible (%)	9.3	21.3	6.3	<0.001
Above-ankle amputation during follow-up (%)	8.1	23.4	4.2	<0.001

Data are expressed as percentages (%) or median ± IQR

*p* value > 0.1 is expressed as n.s

**Table 2:** Identified possible causes why revascularisation, angiography or vascular assessment by a vascular surgeon was not performed within patients with TcPO_2_ < 25 mmHg (*n* = 25)

Identified possible causes	Patients (*n*)
Performed vascular duplex, but were not evaluated further.	5
Healed ulcer	8
Severe renal impairment	4
Severe comorbidity	1
Patients died before evaluation	1
Unknown reasons	6

**Fig. 1:**
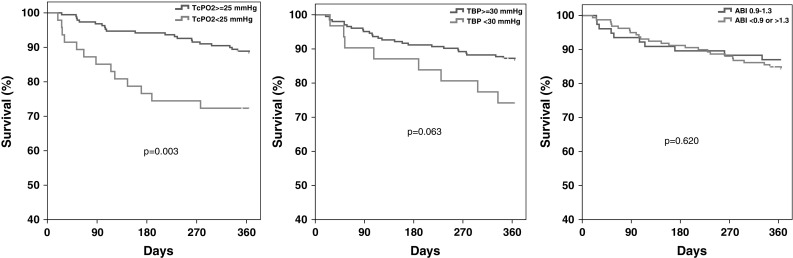
Kaplan–Meier survival curve with log-rank test in DFU patients grouped according to **a** TcPO_2_ < 25 mmHg and ≥ 25 mmHg, *p* = 0.003, **b** TBP < 30 mmHg and ≥ 30 mmHg, *p* = 0.063 and **c** ABI 0.9–1.3 and < 0.9 or > 1.3, *p* = 0.620

**Fig. 2:**
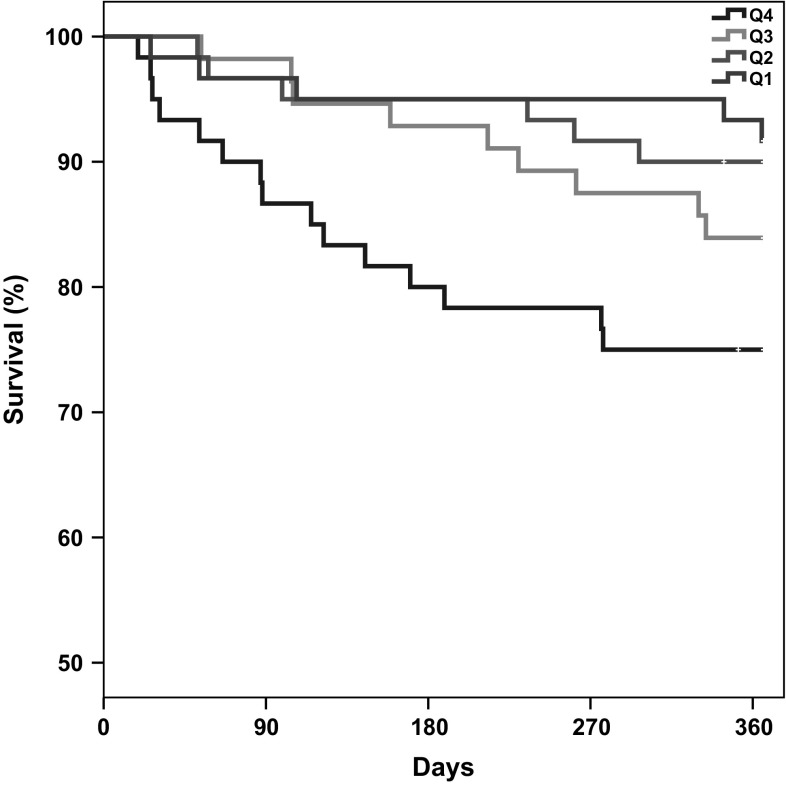
One-year mortality (%) in patients divided into different quartiles of TcPO_2_ and HR calculated for each quartile. Q1 (reference group): ≥ 52 mmHg, Q2: 41–51 mmHg, Q3: 29–40 mmHg, Q4: ≤ 28 mmHg. Pooled comparison (log rank) *p* = 0.032. For separate comparisons in a univariate Cox regression model: Q1 versus Q4: HR 3.4 (1.2–9.4), *p* = 0.018. Q1 versus Q3: HR 2.0 (0.67–5.9), ns. Q1 versus Q2: HR 1.2 (0.4–4.0), ns

After 1 year of follow-up, 35 patients were deceased (14.8%). Patients who died within 1 year were significantly older [81 (75–84) vs. 75 (68–82) years old, *p* = 0.005] and had worse renal function, compared to survivors [eGFR 50 (26–66) vs. 63 (46–83) ml min^−1^ 1.73 m^2^, *p* = 0.002]. TcPO_2_ < 25 mmHg was significantly associated with a higher 1-year mortality rate (27.7 vs. 11.6%, *p* = 0.003), as demonstrated in Fig. 1a. The impact of different TcPO_2_ levels on mortality is illustrated in Fig. 2. There was a not significant trend (*p* = 0.061) in the Kaplan–Meier analysis of worse survival rates in patients with TBP < 30 mmHg (Fig. 1b). ABI < 0.9 or > 1.3 was not linked to a higher mortality rate (Fig. 1c). There was a trend towards better 1-year survival among patients with TcPO_2_ < 25 mmHg who underwent a revascularisation procedure (91.7 vs. 65.7%, in patients not re-vascularised, *p* = 0.094). To adjust for confounders, a Cox proportional analysis was performed. The following factors (age, gender, eGFR, 3 months ulcer healing, revascularisation and above-ankle amputation), that either differed, or nearly differed, between groups at baseline, or was significantly associated with mortality in a univariate analysis were entered stepwise into a multivariate Cox model, together with our variables of interest. Of these plausible confounding factors, only age and renal function (eGFR) were significantly predicting mortality, and thus kept in the final model together with TcPO_2_ and TBP. ABI, revascularisation and ulcer healing at 3 months did not predict mortality or were significant confounders, and consequently, not entered in the final Cox analysis. There were a few cases of missing data, and these were consequently censored in the Cox analysis (*n* = 2 in ulcer healing at 3 months and *n* = 2 in eGFR). The result of the final multivariate Cox analysis is given in Table 3, and as shown, a TcPO_2_ < 25 mmHg was an independent predictor for 1-year mortality with a HR of 2.8 (95% CI 1.34–5.91, *p* = 0.006). Also, when analysing TcPO_2_ as a continuous variable, a significant association between increased survival with each mmHg increasing TcPO_2_ level was found. TBP was not an independent predictor for mortality.

**Table 3:** Results of the Cox regression models with TcPO_2_ and TBP analysed first as continuous and then as dichotomised variables

	HR (95%CI)	95% CI	*p* value
TcPO_2_ (continuous)^a^	0.979	0.959–0.999	0.039
TcPO_2_ < 25 mmHg^a^	2.814	1.341–5.905	0.006
Age^a^	1.061	1.010–1.115	0.018
eGFR^a^	0.984	0.969–0.999	0.034
TBP (continuous)^a^	1.001	0.989–1.012	n.s
TBP < 30 mmHg^a^	1.240	0.526–2.923	n.s
ABI (continuous)	0.623	0.248–1.568	n.s
ABI < 0.9 or > 1.3	0.831	0.399–1.730	n.s

^a^Variables entered in the final multivariate model

## Discussion

TcPO_2_ has in several studies been associated with ulcer healing and has in previously published studies been associated with cardiovascular events and mortality in patients with type 2 diabetes, but without DFU and/or a history of previous CVD [11, 12, 15–17]. Our study is the first to demonstrate that TcPO_2_ also may be a predictor for mortality in the high-risk population of patients with DFUs, with a 2.8-folded increase in 1-year mortality among patients with TcPO_2_ < 25 mmHg. We demonstrated a continuous relation between TcPO_2_ levels and survival, with increasing survival for each mmHg higher TcPO_2_. Today, the current consensus among experts is that ulcer healing more likely occurs when TcPO_2_ ≥ 25 mmHg and TBP ≥ 45 mmHg, and urgent vascular imaging should be considered when TcPO_2_ < 25 mmHg or TBP < 30 mmHg [10]. In our study, 91.8% of our patients with baseline TcPO_2_ < 25 mm Hg failed to heal their ulcers in 3 months, a result similar with others. In the study by Pecoraro et al. [18], there was a 39-fold increase in healing failure if TcPO_2_ was < 20 mmHg. Kalani et al. [15], evaluated the threshold of TcPO_2_ < 25 mmHg, and found impaired healing in 31 out of 34 ulcers (91.2%), compared to improvement in ulcer area in 34 of 37 patients with TcPO_2_ ≥ 25 mmHg. In the Kalani study, ulcer improvement seemed to be more prevalent, compared with our results, but as they evaluated improvement (defined as a decrease in ulcer area of 25%) after 12 months, instead of complete epithelisation after 3 months, as we did, it is difficult to compare these results with ours [15]. In another study, evaluating hyperbaric oxygen therapy and wound healing, no ulcer healed when TcPO_2_ was < 25 mmHg [16].

We observed a high rate of above-ankle amputations (23.4%) in the group of people with TcPO_2_ < 25 mmHg (median TcPO_2_ 11 (5–22) mmHg). Faglia et al. [17] reported major amputation rates of 9.8% in their entire cohort, consisting of patients with PVD (defined as ankle pressure < 70 mmHg, TcPO_2_ < 50 mmHg or obstruction identified at duplex scanning). In their study, the prediction probability of above-the-ankle amputation correlated to the level of TcPO_2_ (after revascularisation), with a probability of 2.6, 6.1, 22.5, 44.0 and 68.0% when TcPO_2_ was 40–49, 30–39, 20–29, 10–19 and < 10 mmHg, respectively. We did only report baseline TcPO_2_, which is a limitation, and reported a total amputation rate of 8.1% in our entire cohort, with a median TcPO_2_ of 41 (28–52). In a separate analysis of patients with TcPO_2_ < 50 mmHg (*n* = 174), the rate increased to 9.8%, similar to the result by Faglia et al. Patients who undergo major amputations are considered to even higher mortality rates, but neither above-ankle amputations nor healing failure was associated with 1-year mortality or was confounding the predictive value of TcPO_2_ on mortality, in our study [19–21]. TBP or ABI were not significantly associated with mortality in our study, which is in contrast to previous results. In a study by Zobel et al. [22], a significant association between toe–brachial index (TBI), ABI and mortality was found. Our cohorts differed, however, in baseline criteria as they included patients with type 2 diabetes and microalbuminuria, but no clinical signs of CVD, compared to our unselected high-risk population of DFU patients. Further, follow-up time was 6 years in the study by Zobel, compared to 1 year in our study. Therefore, one can speculate on whether the trend towards worse outcome seen among patients with TBP < 30 mmHg, would have significantly affect mortality in a longer perspective. Another study, including 81 type 2 diabetes with a history of myocardial infarctions, evaluated the risk of recurrent MACE when TBP < 50 mmHg and reported a HR of 3.83 (1.45–10.1) [23]. Further, in the Hoorn study, a population-based cohort study, including 155 patients with, and 469 patients without type 2 diabetes, Hanssen et al. [24] demonstrated a significant association between ABI < 0.9 and all-cause mortality in both patients with and without diabetes, after a median follow-up time of 17.2 years. Our present study, evaluating 1-year mortality in the high-risk population of patients with type 2 diabetes and DFU indicates that TcPO_2_ might be a useful tool for risk assessment, with a prognostic value in the short-term perspective, superior compared to ABI and TBP.

Concerning revascularisation, the observed rate of 25.5% among patients with TcPO_2_ < 25 mmHg is considerably low. Additional 21.3% performed diagnostic imaging and were assessed by a vascular surgeon, but were not amenable for revascularisation. More than half of the patients with TcPO_2_ < 25 mmHg did not, however, perform a complete diagnostic assessment, which is inconsistent with the recommendation by IWGDF. When evaluating those cases separately, we found cases with prompt ulcer improvement, as well as patients with severe comorbidity, including renal failure, among these individuals, but also a few cases with unknown reasons were limb ischaemia with possibility for revascularisation might have been under-diagnosed. Patients with TcPO_2_ < 25 mmHg who underwent revascularisation procedures had a trend towards better survival, but in the 1-year perspective this finding did not reach significance, or was confounding our results. In a study by Faglia et al. [21], patients with identified critical limb ischaemia had higher survival rates after successful intervention, during 6 years of follow-up. However, when the authors adjusted for confounders in a Cox model, only age turned out to predict mortality in that study [21]. Our low revascularisation rate is, however, an important observation, but not unique in our setting. In a German study by Malyar et al. [25], only 18% of the patients were re-vascularised, and another 25% were evaluated with angiography. Also, in the EURODIALE study, vascular imaging was only performed in 56% of patients with severe limb ischaemia, and of them, only 43% were re-vascularised [26]. This might reflect the complexity of patients with DFUs, where several factors, and often severe comorbidity need to be considered before intervention. Nevertheless, further optimising the multidisciplinary approach, when managing DFU patients, is desirable.

We can only speculate about the mechanisms behind the increase in all-cause mortality, among our patients with low TcPO_2_. Traditionally cardiovascular risk factors, such as smoking, hypertension and previous CVD, did not significantly relate to either TcPO_2_ or mortality, in our study. One possible explanation could be that a low TcPO_2_ on the foot might serve as a marker for impaired microcirculation in general, with a higher burden of microvascular complications. It has previously been shown by Huang et al. [27], a significant negative association between TcPO_2_ and microvascular events (albuminuria and distal polyneuropathy) in type 2 diabetes patients, with a tenfold risk of microvascular complications when TcPO_2_ was < 50 mmHg. Similar, our study demonstrated a significant association between a low TcPO_2_ and renal impairment, and a low eGFR did also independently associated with mortality, results previously shown by many [28–30]. eGFR did not, however, confound the impact of TcPO_2_ on mortality in the Cox analysis. Another possible mechanism contributing to the increased mortality rate among patients with low TcPO_2_, could be a higher prevalence of neuropathy [31]. In a study by Arora et al. [32], a lower TcPO_2_ in the forearm and foot was found in patients with diabetic neuropathy, compared to non-neuropathic patients, as well as healthy subjects. Another study by Pfutzner et al. [33] indicated a relation between microvascular disturbance and small fibre neuropathy. As small fibre neurons are involved both in peripheral neuropathy, and in the serious diabetes complication cardiac autonomic neuropathy, the latter could be a plausible mechanism contributing to increased mortality in patients with microvascular impairment [34]. In our study, we lack quantitative data on neuropathy, and thus, we can only speculate on a causality.

Our study has some strengths and limitations that need to be stated. The study design as a retrospective cohort study might be afflicted with bias, and the findings might only serve as hypothesis generating for future prospective studies. However, one strength is our relatively large cohort size, which is representative of the DFU-population as most patients with DFUs in our region are transferred to our multidisciplinary DFU-unit. Further, the vast majority of our patients are routinely screened with TcPO_2_, TBP and ABI and are seen at a regularly basis at our department, until ulcer healing. Mortality data are accurate since all deaths in Sweden are registered in our National Death Register. We only report all-cause mortality, due to the low frequency of autopsies in Sweden, and thus, we can thus only speculate on underlying cause of death. The lack of quantitative data on neuropathy and albuminuria is a limitation, and the association between TcPO_2_ and cardiac autonomic neuropathy needs further studies. Several factors, besides microvascular function, might contribute to a low TcPO_2_ that we did not correct for, such as advanced pulmonary diseases or heart failure with chronic hypoxia, as well as local factors such as tissue oedema and inflammation. Only one patient in the TcPO_2_ < 25 mmHg group was suffering from chronic obstructive pulmonary disease, but since we did not measure oxygen saturation, undiagnosed hypoxia could have affected our results.

Besides limitations, our novel findings indicate an independent association between a low TcPO_2_ and increased 1-year mortality in patients with type 2 diabetes and DFU. This suggests that screening with TcPO_2_ might improve identification of DFU patients with urgent need for both vascular intervention and intensive cardiovascular risk factor assessment.

## Abbreviations

ABI: Ankle–brachial index
CVD: Cardiovascular disease
DFU: Diabetic foot ulcer
eGFR: Estimated glomerular filtration rate
HR: Hazard ratio
IWGDF: The International Working Group on the Diabetic Foot
IQR: Interquartile range
MACEs: Major cardiovascular events
MDRD: Modification of diet in renal disease
PVD: Peripheral vascular disease
TcPO_2_: Transcutaneous oxygen pressure
TBP: Toe blood pressure
